# Real-World Experience With Collagenase Clostridium Histolyticum-aaes for Buttock and Thigh Cellulite: Focus on Administration and Safety Profile

**DOI:** 10.1093/asjof/ojad014

**Published:** 2023-02-20

**Authors:** Sachin M Shridharani, MacKenzie L Kennedy

## Abstract

**Background:**

Collagenase clostridium histolyticum-aaes (CCH-aaes) is approved for the treatment of moderate-to-severe buttock cellulite in adult women.

**Objectives:**

To report on real-world experience with CCH-aaes in the treatment of buttock and thigh cellulite.

**Methods:**

Retrospective analysis of medical records from a single treatment center.

**Results:**

The population comprised 28 consecutively treated women; mean age was 40.5 years (range, 23-56 years) and mean body mass index was 25.9 kg/m^2^ (range, 19.6-41.0 kg/m^2^). Treatment areas were buttocks only (78.6% of patients), thighs only (10.7%), or both buttocks and thighs (10.7%). Most patients (89.3%) were treated in 2 areas (buttocks or thighs) at each visit; however, 3 patients were treated in 4 areas. At each session, the CCH-aaes dose was 0.07 mg per dimple (0.3 mL of 0.23 mg/mL for buttock cellulite; 1.5 mL of 0.046 mg/mL for thigh cellulite). The mean number of treatment sessions was 2.6 (range, 1-4) for buttock cellulite and 2.5 (range 1-3) for thigh cellulite. The mean number of dimples treated was 11.5 (range, 3-17) per buttock, 11.0 (range, 1-14) per thigh, and 23.4 (range, 8-32) overall per treatment session. Injection site–related adverse events of special interest were experienced by all 28 patients: bruising (100%), edema (96.4%), tenderness (85.7%), nodules (39.3%), pruritus (32.1%), and hyperpigmentation indicative of hemosiderin staining (7.1%). Mean duration of injection-site bruising was 8.8 days (range, 2-15 days).

**Conclusions:**

CCH-aaes is an effective, well-tolerated, minimally invasive treatment option for buttock and thigh cellulite in women.

**Level of Evidence: 3:**

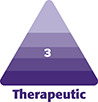

##  

Cellulite affects 80% to 98% of postpubertal females and is a major cosmetic concern for many women.^1–4^ Women seeking treatment for cellulite may consult plastic surgeons, dermatologists, or other aesthetic medicine professionals. Interventions intended to minimize the appearance of cellulite include topical agents, mechanical stimulation, energy-based devices (eg, laser, light, and radiofrequency), acoustic wave or acoustic pulse therapy, dermal fillers, and subcision.^2,5–7^

Collagenase clostridium histolyticum-aaes (CCH-aaes; Qwo, Endo Aesthetics LLC, Malvern, PA) is composed of 2 purified bacterial collagenases (AUX-I and AUX-II [clostridial class I and II collagenases]) that hydrolyze types I and III collagen with high specificity.^8,9^ Injection of CCH-aaes breaks down mature, collagen-rich (fibrous) septa and stimulates the formation of new (immature) collagen and redistribution of fat lobules to produce a smoother skin contour.^10^ CCH-aaes is approved by the US Food and Drug Administration as a subcutaneous injection for the treatment of moderate-to-severe buttock cellulite in adult women.^8^ The efficacy and safety of CCH-aaes for buttock cellulite has been demonstrated in 3 randomized placebo-controlled trials^11–13^ and an open-label extension trial,^14^ and the product labeling includes instructions for use.^8^ Although some trials enrolled women with posterolateral thigh cellulite,^11,14^ there is limited published information regarding injection technique,^15^ study data,^16^ or clinical experience^17^ with off-label use of CCH-aaes for thigh cellulite. The goal of this study was to report on real-world experience with CCH-aaes in the treatment of buttock and thigh cellulite.

## METHODS

This study was a retrospective analysis of medical records from a single treatment center and included all females treated with CCH-aaes for buttock and/or thigh cellulite between March 2021 and July 2022. Deidentified patient information was extracted from medical records into a database that contained no links to patient identifiers; therefore, the study was exempt from institutional review board approval. All patients provided written informed consent for the use of deidentified information from their medical records.

Cellulite treatment areas included buttocks and/or thighs and prior to each session, dimples were marked for treatment. CCH-aaes is available as a lyophilized powder that is reconstituted using a supplied diluent.^8^ For the treatment of buttock cellulite, CCH-aaes was reconstituted as recommended in the product label (0.92 mg with 4 mL diluent or 1.84 mg with 8 mL diluent), for a concentration of 0.23 mg/mL after reconstitution. For each buttock dimple treated, one 0.3-mL injection of CCH-aaes was administered in three 0.1-mL aliquots (Video). Each syringe was loaded with 0.9 mL (0.23 mg/mL) of CCH-aaes to allow for 3 buttock injections per syringe.

**Figure 1.**
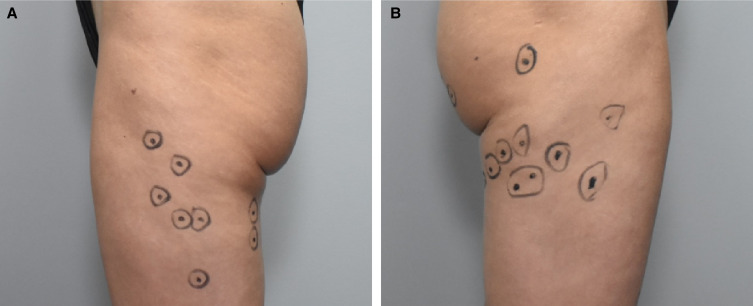
A 38-year-old female patient with dimples marked for CCH-aaes injection in the (A) left and (B) right thigh. CCH-aaes, collagenase clostridium histolyticum-aaes.

CCH-aaes for the treatment of thigh cellulite was reconstituted using a greater dilution than that for buttock cellulite: 1.84 mg with 8 mL diluent and 32 mL of preservative-free normal saline, for a concentration of 0.046 mg/mL after reconstitution. Figure 1 shows a patient with thigh dimples marked for CCH-aaes injection. For each thigh dimple treated, one 1.5-mL injection of CCH-aaes was administered (in five 0.3-mL aliquots). Each syringe was loaded with 3 mL (0.046 mg/mL) of CCH-aaes to allow for 2 thigh dimple injections per syringe. Use of the greater dilution and greater volume of administration for thigh cellulite in this study provided the same dose of CCH-aaes per dimple (0.07 mg) that is recommended for buttock cellulite in the US prescribing information.^8^ The dilution and administration procedures for thigh cellulite were consistent with results from a 2022 study of CCH-aaes injection techniques.^15^ Post-marking photography was referenced at subsequent treatment sessions. Any dimples still present were marked for additional CCH-aaes treatment, and other dimples (if present) also may have been marked for initial treatment.

Information on patient demographics, history of cosmetic procedures, CCH-aaes treatment, and adverse events (AEs) was extracted from each medical record and summarized using descriptive statistics. AEs obtained from patient reports and/or physician examination were captured in each patient's medical record. AE extraction for this study included the following AEs of special interest for CCH-aaes: bruising, edema, nodules, pruritus, tenderness, and hyperpigmentation (considered indicative of hemosiderin staining, although a biopsy to confirm diagnosis of hemosiderin staining was not performed). Bruising was considered mild in intensity, regardless of the degree of discoloration, if it did not interfere with patient daily activities or require therapeutic intervention (eg, analgesic medication).

## RESULTS

A total of 28 consecutively treated women were included in this study (Table). Patient age ranged from 23 to 56 years (mean, 40.5 years), with 13 women (46.4%) aged <40 years. Twelve patients (42.9%) had normal body mass index, and the remainder were overweight or obese. A history of nonsurgical cosmetic procedures was reported by 17 patients (60.7%) and a history of cosmetic surgery by 16 patients (57.1%). Eleven women (39.3%) had previously received both nonsurgical and surgical cosmetic procedures, whereas 6 (21.4%) were naïve to cosmetic procedures at the time of the first CCH-aaes treatment session. One patient had received a previous treatment for cellulite (ultrasound). In the current study, patients were treated with CCH-aaes for cellulite in the buttocks only (78.6%), thighs only (10.7%), or buttocks and thighs (10.7%; Table). Of the 6 patients treated for thigh cellulite, the treatment area was posterior thighs in 4 patients, anterior thighs in 1 patient, and both anterior and posterior thighs in 1 patient. The mean duration of follow-up from the date of the first CCH-aaes treatment was 35.9 weeks (range, 6.1-75.3 weeks).

**Table:** Demographic and Baseline Characteristics

Parameter	Patients (*n* = 28)
Female, *n* (%)	28 (100)
Age, y, mean ± SD (range)	40.5 ± 8.8 (23-56)
Race, *n* (%)	
White	25 (89.3)
Black	2 (7.1)
Asian	1 (3.6)
Hispanic/Latino, *n* (%)	7 (25.0)
Fitzpatrick skin type, *n* (%)	
II	9 (32.1)
III	11 (39.3)
IV	5 (17.9)
V	3 (10.7)
BMI, kg/m^2^, mean ± SD (range)	25.9 ± 4.6 (19.6-41.0)
BMI category, *n* (%)	
Normal (18.5 to <25 kg/m^2^)	12 (42.9)
Overweight (25 to <30 kg/m^2^)	12 (42.9)
Obese (≥30 kg/m^2^)	4 (14.3)
Location of cellulite treated, *n* (%)	
Buttock only	22 (78.6)
Thigh only	3 (10.7)
Buttock and thigh	3 (10.7)
Prior nonsurgical cosmetic procedures, *n* (%)	17 (60.7)
Injectables: fillers	13 (46.4)
Injectables: botulinum toxin type A	7 (25.0)
Injectables: deoxycholic acid (Kybella; Allergan, Madison, NJ)	2 (7.1)
Laser (Sciton, Palo Alto, CA)	2 (7.1)
Cellulaze (Cynosure, Westford, MA)	1 (3.6)
CoolSculpting (Allergan Aesthetics, Pleasanton, CA)	1 (3.6)
Injectables: poly-L-lactic acid (Sculptra; Galderma Laboratories, Fort Worth, TX)	1 (3.6)
Microneedling	1 (3.6)
Ultrasound cellulite treatment	1 (3.6)
VelaShape (Candela Medical, Marlborough, MA)	1 (3.6)
Prior surgical cosmetic procedures, *n* (%)	16 (57.1)
Liposuction	9 (32.1)
Breast augmentation/breast implant	7 (25.0)
J-plasma device (Apyx Medical, Clearwater, FL)	6 (21.4)
Rhinoplasty/septoplasty/nose reconstruction	5 (17.9)
Abdominoplasty	4 (14.3)
BBL/fat transfer to buttock	2 (7.1)
Mastopexy	2 (7.1)
Otoplasty (bilateral, external ear)	1 (3.6)
Recti diastasis repair	1 (3.6)
Skin removal	1 (3.6)

BBL, Brazilian butt lift; BMI, body mass index; SD, standard deviation.

The mean CCH-aaes dose administered at a single treatment session was 1.61 mg (range, 0.55-2.21 mg), with a mean of 23.4 dimples treated (range, 8-32). The maximum CCH-aaes dose administered at any treatment session exceeded the recommended maximum of 1.68 mg in 5 patients: 4 received 1.79 mg (ie, 26 dimples treated), and 1 received 2.21 mg (ie, 32 dimples treated). The patient who received a total CCH-aaes dose of 2.21 mg was treated in both buttocks and both thighs (4 treatment areas) after 2 previous visits in which treatment of thigh cellulite with a CCH-aaes dose up to 1.38 mg (total for both thighs) was well tolerated.

For most patients (89.3%), 2 areas (buttocks or thighs) were treated at each visit; however, 2 patients were treated in both buttocks and both thighs (4 areas) at one visit each, and 1 patient was treated in both anterior and posterior thighs (4 areas) at 3 visits. For buttock cellulite, the mean number of CCH-aaes treatment sessions was 2.6 (range, 1-4), with most patients (16/25; 64.0%) receiving 3 sessions that were separated by ∼3 weeks, as recommended in US prescribing information.^8^ Across treatment sessions, the mean number of dimples treated was 11.5 (range, 3-17) per buttock, and the mean CCH-aaes dose administered was 0.79 mg (range, 0.21-1.17 mg) per buttock. For thigh cellulite, the mean number of treatment sessions was 2.5 (range, 1-3). Across treatment visits, the mean number of dimples treated was 11.0 (range 1-14) per thigh; mean CCH-aaes dose administered was 0.76 mg (range, 0.07-0.97 mg) per thigh.

All 28 patients experienced injection site–related AEs: bruising (100% [28/28]), edema (96.4% [27/28]), tenderness (85.7% [24/28]), nodules (39.3% [11/28]), pruritus (32.1% [9/28]), and hyperpigmentation indicative of hemosiderin staining (7.1% [2/28]). Of 78 total treatment sessions across the patient population, AEs were reported after 76 sessions (97.4%). All AEs were considered by the clinician to be mild in intensity, and no serious AEs were reported.

**Figure 2.**
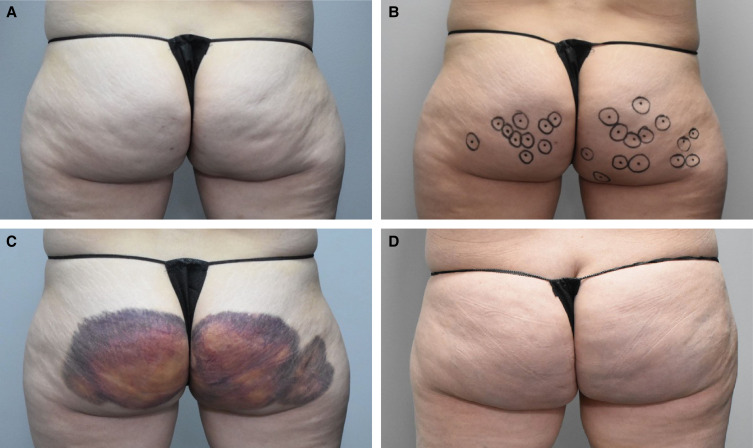
A 40-year-old female at (A) baseline, (B) with dimples marked for CCH-aaes injection, (C) with bruising and edema 3 days after the first treatment session with CCH-aaes in the buttocks, and (D) after resolution of bruising. CCH-aaes, collagenase clostridium histolyticum-aaes.

**Figure 3.**
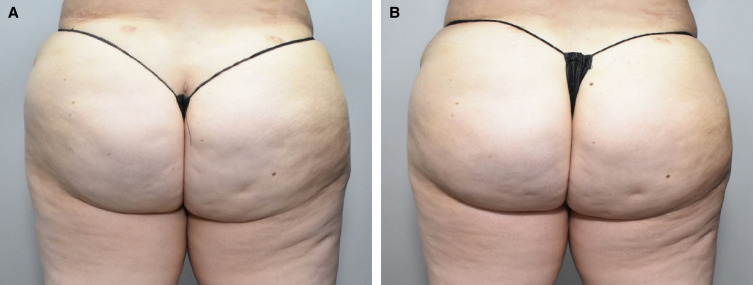
A 32-year-old female at (A) baseline and (B) 22 days after the third treatment session with CCH-aaes in the buttocks. CCH-aaes, collagenase clostridium histolyticum-aaes.

Injection-site bruising occurred after 70 of 78 treatments (89.7%). Figure 2 shows the markings for CCH-aaes injections and bruising pattern after the first treatment session in 1 patient. In 5 patients who had experienced injection-site bruising after the first and second treatment sessions, there was no bruising after the third CCH-aaes treatment session (Figure 3). The mean duration of injection-site bruising was 8.8 days (range, 2-15 days) overall. Mean bruising duration decreased over the course of CCH-aaes treatment: 10.9 days after the first session, 7.5 days after the second session, and 6.5 days after the third session. In the 2 patients (both Fitzpatrick skin type IV) with hyperpigmentation indicative of hemosiderin staining, this AE resolved after ∼7 weeks in 1 patient and after ∼3.5 months in the other patient. There was no apparent relationship between patient age or body mass index and AEs of special interest, including for the most common AE of injection-site bruising. Treatment cost and injection-site bruising were the most common reasons that patients chose to receive fewer than 3 treatment sessions.

Among 11 patients with no previous nonsurgical cosmetic procedures and 12 patients with no previous cosmetic surgery (6 of these patients were naïve to both), 1 patient received an additional cosmetic procedure within 12 months of initiating CCH-aaes. Two patients, who had previously received liposuction, received mini thigh lifts after completing CCH-aaes treatment for buttock and thigh cellulite (1 patient) or buttock cellulite (1 patient).

## DISCUSSION

The focus of this case series was to present real-world experience with CCH-aaes for the treatment of cellulite in the buttocks and thighs. While administration of CCH-aaes was typically consistent with the US product label, variations in our clinical practice included treatment of >2 areas at a treatment session (up to 4 areas) and >12 injections administered per treatment area (up to 17 injections per area). When >2 areas were treated (or >12 injections were administered per treatment area), the CCH-aaes volume and concentration per injection (0.3 mL of 0.23 mg/mL for buttock cellulite, 1.5 mL of 0.046 mg/mL for thigh cellulite), time interval between treatment sessions (∼3 weeks), and post-treatment care were unchanged. With one exception, the total dose of CCH-aaes administered during a single treatment session did not exceed 1.79 mg (slightly higher than the 1.68 mg recommended in the US product label). Results of this study show that CCH-aaes treatments involving >2 areas and dosing somewhat higher than recommended were generally safe and well tolerated. In addition, although the sample size was small, no new safety signals were observed with off-label use of CCH-aaes (anterior as well as posterior thigh cellulite).

Compared with the dosing and injection procedure for buttock cellulite (0.3 mL [0.23 mg/mL] administered in three 0.1-mL aliquots per dimple), a study evaluating CCH-aaes injection techniques suggested that treatment outcomes for thigh cellulite might be optimized by using a different dilution and injection technique: 1.5 mL (0.047 mg/mL) administered in five 0.3-mL aliquots per dimple, using both deep (1-inch) and shallow (1/2-inch) needle positioning.^15^ These recommendations for dilution and volume of administration were used for CCH-aaes treatment of thigh cellulite in the current report. The CCH-aaes treatment approach used for this study, including areas treated, CCH-aaes dilutions, injection techniques, and number of sessions, is consistent with that suggested by a panel of other clincians.^17^ Of note, the provided diluent is calcium-based and required for activation of CCH-aaes. Further expansion of volume for thigh treatment requires preservative-free normal saline, as the presence of preservative(s) would lead to decreasing potency of the drug.

The AEs of special interest profile observed in this case series was consistent with that reported in randomized controlled trials, with injection-site bruising being the most commonly observed AE.^12^ Although injection-site bruising occurred in all patients after the first CCH-aaes treatment session in the current study, it tended to lessen with subsequent treatments, and some patients had no bruising following the third treatment session. In this study, bruising resolved within ∼1 week on average (within 15 days in all patients), and the duration of bruising tended to decrease with each successive CCH-aaes administration. Bleeding is not thought to be a direct effect of the drug itself. While CCH-aaes cleaves types I and III collagen, the basement membrane of blood vessels is composed of type IV collagen, which has been shown histologically to remain intact when CCH-aaes is injected in proximity.^9,10^ With the relatively immediate effect of the CCH-aaes in disrupting the collagen septa, there is often an associated vascular plexus in proximity, typically a thin-walled venule, which ruptures upon release of the dermis from the underlying skeletal musculature previously tethered by the septa. With subsequent treatments occurring within a short interval, the same anatomic area typically has had insufficient time for revascularization and regrowth of the venules. Long-term histologic assessment of tissues treated with CCH-aaes has shown dermal thickening, neocollagenesis, and fat lobule/adipocyte reorganization.^10^

Since the completion of this study, the authors have begun prescribing oral tranexamic acid, beginning the day prior to CCH-aaes injections and continuing through 3 days post-treatment, to mitigate bruising. Other bruising mitigation strategies in clinical use include Radial Soundwave Technology (Zimmier Medizin Systems, Irvine, CA) and pulse dye laser treatments, administered several days after CCH-aaes injection.^17^ Real-time assessment of bruising using a quantitative scale may help clinicians track the occurrence and resolution of this AE. Hyperpigmentation indicative of hemosiderin staining was observed in 2 patients but resolved after several weeks to months. Prior to initiating treatment with CCH-aaes, patients were counseled that bruising after the first treatment and possibly the second treatment may be substantial and that several days to weeks of social downtime may be advisable. Patients were also informed that a small percentage of individuals, for unknown or unpredictable reasons, have prolonged bruising that leads to staining with a rust-like appearance, which seems to resolve but can take upwards of 1 year. The primary management strategy for reducing the risk of hemosiderin staining involves mitigation of bruising. Prolonged bruising, with the presence of hemoglobin leaking into the tissue, leads to hemosiderin staining; thus, reduction in bruising severity may lessen subsequent hemosiderin deposits.

In December 2022, after this study was completed, the manufacturer of CCH-aaes announced that it would cease production and sale of the product due to concerns about the extent and variability of bruising after the initial treatment session and the possibility of prolonged skin discoloration. From a clinical perspective, removing this product from the market has a negative impact on the treatment algorithm for patients interested in management and treatment of cellulite. For one, CCH-aaes served as the only FDA-approved injectable to manage this complex aesthetic condition. This was a new category that provided patients with a treatment option other than a device, which often had limited success, or alternatively, minor surgical management.

Most women who opted for cellulite treatment with CCH-aaes had previously received other surgical and/or nonsurgical cosmetic procedures. For patients who had no such prior experience, interest in cellulite treatment prompted their initial contact with a plastic surgeon, and experience with CCH-aaes, which is relatively non-invasive, may increase their comfort with pursuing additional cosmetic procedures to address other issues. Limitations of the study were that it was a single-center observation, the analysis was retrospective, the severity of cellulite was not recorded, and treatment efficacy was not evaluated formally.

Objective measurement of cellulite severity and treatment efficacy in the clinic is hindered by the lack of a tool that is both validated and convenient to use.^18,19^ The Patient Reported Photonumeric Cellulite Severity Scale and Clinician Reported Photonumeric Cellulite Severity Scale have been used in randomized controlled trials of CCH-aaes.^11,12^ However, they require either live assessments or specialized equipment and setup (eg, camera settings, lighting conditions, patient positioning) to enable collection of high-quality photographs using standardized procedures,^19^ which were not available for this study.

## CONCLUSIONS

In this real-world study, CCH-aaes was a well-tolerated, minimally invasive treatment option for buttock and thigh cellulite in women. The study provides further insights into aspects of CCH-aaes treatment (eg, for thigh cellulite, treating >2 areas in 1 session, and/or >12 dimples per area).

## Supplementary Material

ojad014_Supplementary_Data
